# Supplementary material for: Modelling safe protocols for reopening schools during the COVID-19 pandemic in France
Source: Nat Commun. 2021 Feb 16;12:1073. doi: 10.1038/s41467-021-21249-6 (PMC7887250; doi:10.1038/s41467-021-21249-6)
Supplement: Supplementary file 1 — Supplementary Information [file 41467_2021_21249_MOESM1_ESM.pdf]

SUPPLEMENTARY INFORMATION

Modelling safe protocols for reopening schools during the COVID-19 pandemic in France

Laura Di Domenico[1], Giulia Pullano[1,2], Chiara E. Sabbatini[1], Pierre-Yves Boëlle[1], Vittoria Colizza[1,3,*]

1 INSERM, Sorbonne Université, Pierre Louis Institute of Epidemiology and Public Health, Paris, France
2 Orange Labs, Sociology and Economics of Network and Services (SENSE), Chatillon, France
3 Tokyo Tech World Research Hub Initiative, Institute of Innovative Research, Tokyo Institute of Technology, Tokyo, Japan

* vittoria.colizza@inserm.fr

Supplementary Note 1: Compartmental model 2

1.1. Structure of the compartmental model 2

1.2. Generation time distribution 3

1.3. Estimation of within-hospital parameters 4

Supplementary Note 2: Model calibration 6

Supplementary Note 3: Model parametrization during lockdown and exit phase 7

Supplementary Note 4: Additional results 8

4.1. Additional results corresponding to values of younger children relative transmissibility not shown in main paper 8

Supplementary Note 5: Sensitivity analysis 10

5.1. Results obtained with an additional set of scenarios 10

5.2. Results obtained with 10% variations of the reproduction number during lockdown 12

5.3. Results obtained with moderate interventions and 25% case isolation 13

5.4. Results obtained with 50% reduction of contacts engaged by adolescents 14

Supplementary References 16

Supplementary Note 1: Compartmental model

1.1. Structure of the compartmental model

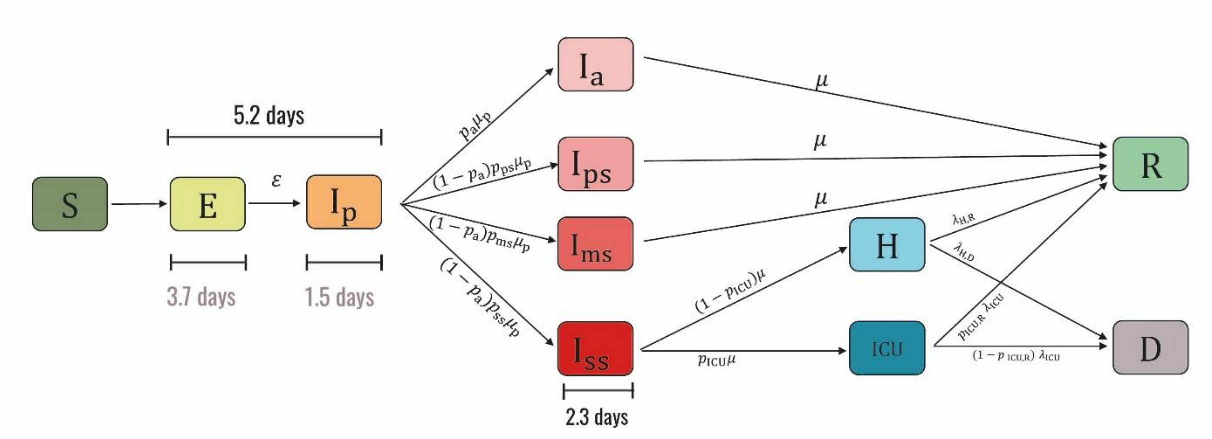

Supplementary Figure 1. Compartmental model. S=Susceptible, E=Exposed, I_p= Infectious in the prodromic phase (the length of time including E and I_p stages is the incubation period), I_a=Asymptomatic Infectious, I_{ps}=Paucysymptomatic Infectious, I_{ms}=Symptomatic Infectious with mild symptoms, I_{ss}=Symptomatic Infectious with severe symptoms, ICU=severe case admitted to ICU, H=severe case admitted to the hospital but not in intensive care, R=Recovered, D=Deceased.

Supplementary Table 1. Parameters, values, and sources used to define the compartmental model[1,2]

Variable	Description	Value	Source
θ^{-1}	Incubation period	5.2d	[3]
μ_{p}^{-1}	Duration of prodromal phase	1.5d, computed as the fraction of pre-symptomatic transmission events out of pre-symptomatic plus symptomatic transmission events	[4]
ϵ^{-1}	Latency period	$\theta^{-1} - \mu_{\mathrm{p}}^{-1}$	-
p_{a}	Probability of being asymptomatic	0.4	[5]
p_{ps}	If symptomatic, probability of being paucisymptomatic	1 for younger children, adolescents<br>0.2 for adults, seniors	[6]
p_{ms}	If symptomatic, probability of developing mild symptoms	0 for younger children, adolescents<br>0.76 for adults<br>0.53 for seniors	[6–8]
p_{ss}	If symptomatic, probability of developing severe symptoms	0 for younger children, adolescents<br>0.04 for adults<br>0.27 for seniors	[7,8]
g	Generation time	6.6d	[9]
μ^{-1}	Infectious period for I_{a}, I_{ps}, I_{ms} , I_{ss}	2.3d, chosen accordingly to generation time distribution (see following section)	-
r_β	Relative infectiousness of I_{p}, I_{a}, I_{ps}	0.1, 0.25, 0.33, 0.55 for younger children<br>0.55 for adolescents, adults, seniors	[10]
s	Relative susceptibility	0.5 for younger children, adolescents<br>1 for adults, seniors	[11]
p_{ICU}	If severe symptoms, probability of going to ICU	0.24 for adults<br>0.24 for seniors	[12]
$\lambda_{\mathrm{H,R}}$	If hospitalized, daily rate entering in R	0.083 for adults<br>0.033 for seniors	[12]
$\lambda_{\mathrm{H,D}}$	If hospitalized, daily rate entering in D	0.0031 for adults<br>0.0155 for seniors	[12]
$p_{\mathrm{ICU,R}}$	Probability of recovery from ICU	0.76 for adults<br>0.54 for seniors	[12]
$\lambda_{\mathrm{ICU}}^{-1}$	Time spent in ICU	21.1d for adults<br>20.7d for seniors	[12]

1.2. Generation time distribution

The generation time distribution in a compartmental epidemic model can be computed thanks to the theory developed by Svensson[13]. Let X and Y be the random variables describing the latency period and the infectious period, respectively. Then the distribution of the generation time is the result of the convolution $f * h_{\mathrm{s}}$, with f being the probability density function of X and

$$h_{\mathrm{s}}(t) = \frac{1 - H(t)}{E(Y)}$$

where H is the cumulative distribution function of Y, and $E(Y)$ is the mean.
In the compartmental model under consideration (Supplementary Figure 1), we have that X is exponentially distributed with rate ϵ, and Y is the sum of two exponentially distributed

random variables (prodromic phase and infectious period, with rate μ_{p} and μ respectively). Computations show that the corresponding generation time distribution is

$$g(t) = \frac{\epsilon\,\mu_{\mathrm{p}}\,\mu}{(\mu_{\mathrm{p}}+\mu)(\mu-\mu_{\mathrm{p}})}\,\Big[\frac{\mu}{(\epsilon-\mu_{\mathrm{p}})}\,(e^{-\mu_{\mathrm{p}}t}-e^{-\epsilon\,t})-\frac{\mu_{\mathrm{p}}}{(\epsilon-\mu)}\,(e^{-\mu\,t}-e^{-\epsilon\,t})\Big]$$

Given the values of ϵ and μ_{p} informed from the literature (Supplementary Table 1), we choose μ so that the mean of the generation time equals to 6.6 days. The shape of the distribution is displayed in Supplementary Figure 2 and it closely resembles a gamma distribution with mean 6.6 and shape parameter 1.87, estimated in Ref.[9]

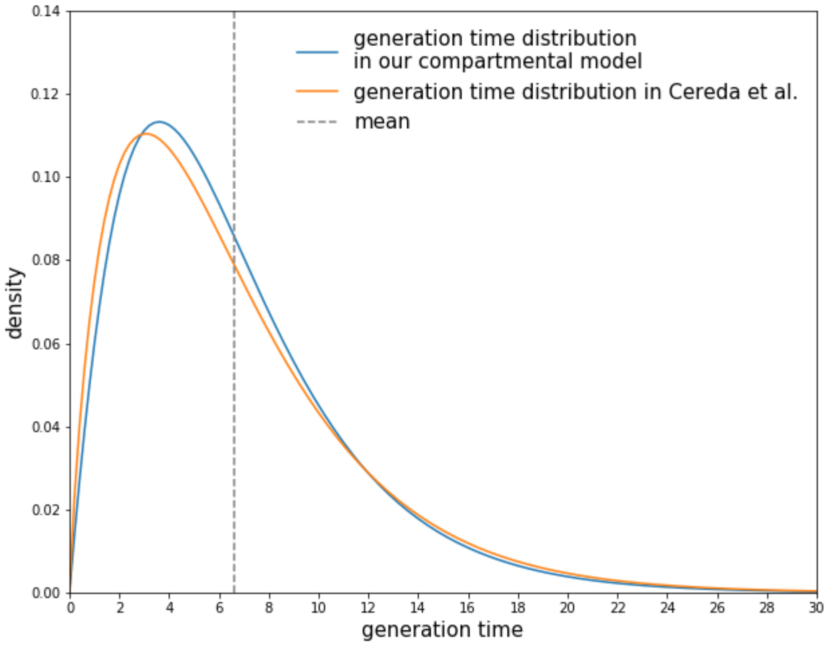

Supplementary Figure 2. Distribution of the generation time. The generation time distribution corresponding to our compartmental model (blue) in comparison with the distribution estimated in Ref.[9] (orange).

1.3. Estimation of within-hospital parameters

We fit data on patient trajectories recorded in Île-de-France hospitals after admission up to April 5, 2020. Data consisted of age, sex, date of hospital admission and subsequent dates of discharge or death, and, when relevant, dates of entering/leaving the ICU. We fit mixture and competing risks models to time to event data, taking into account censoring due to patients being still in the hospital at the time of analysis. We used exponential distributions for time to event data to match the hypotheses of the compartmental epidemic model.

First, we model time from admission to entering the ICU or being discharged/dead for those who do not go to the ICU. Write T for the time to the first of the 3 following events: entering the ICU, being discharged alive or dying in the hospital. T is modelled as a mixture of 2 exponential distributions: $T \sim \pi_{\mathrm{ICU}}\exp(\lambda_{\mathrm{ICU}}) + (1-\pi_{\mathrm{ICU}})\exp(\lambda_{\mathrm{H}})$, where π_{ICU} is the probability to go to the ICU, and λ_{ICU}, λ_{H} are the rates of the exponential distributions. The second exponential describes time spent in the hospital by those who don't go the ICU subject to competition of 2 outcomes, discharge or death. Therefore, $\lambda_{\mathrm{H}} = \lambda_{\mathrm{DIS}} + \lambda_{\mathrm{DTH}}$ where λ_{DIS} is the rate of discharge and λ_{DTH} the rate of death. The average time spent in the hospital is $1/\lambda_{\mathrm{H}}$, and the probability of being discharged alive is $\lambda_{\mathrm{DIS}}/(\lambda_{\mathrm{DIS}}+\lambda_{\mathrm{DTH}})$. Therefore, the likelihood

of a patient trajectory observed up to time t with final status s (comprising still hospitalized - HOS, admitted to ICU - ICU, discharged alive - DIS, dead - DTH) is given by :

$$L(\pi_{\text{ICU}}, \lambda_{\text{ICU}}, \lambda_{\text{DIS}}, \lambda_{\text{DTH}}) = (\pi_{\text{ICU}} \lambda_{\text{ICU}} \exp(-\lambda_{\text{ICU}}\, t))^{s=\text{ICU}}$$

$$\left((1-\pi_{\text{ICU}})\,(\lambda_{\text{DIS}}/(\lambda_{\text{DIS}}+\lambda_{\text{DTH}}))^{s=\text{DIS}}\,(\lambda_{\text{DTH}}/(\lambda_{\text{DIS}}+\lambda_{\text{DTH}}))^{s=\text{DTH}}\exp(-(\lambda_{\text{DIS}}+\lambda_{\text{DTH}})t)\right)^{1-s=\text{ICU}}$$

$$(1-\pi_{\text{ICU}}\exp(-\lambda_{\text{ICU}}\, t) - (1-\pi_{\text{ICU}})\exp(-(\lambda_{\text{DIS}}+\lambda_{\text{DTH}})\, t))^{s=\text{HOS}}$$

The first line is for patients going to the ICU, the second line for those being discharged alive or dead and the third line for patients who were censored because they were still in the hospital.

Likewise, we fit time to discharge or death after admission to the ICU using a competing risk approach with exponential parameters μ for being discharged alive or dead; the likelihood is therefore:

$$L(\mu_{\text{DIS}}, \mu_{\text{DTH}}) =$$

$$\left((\mu_{\text{DIS}}/(\mu_{\text{DIS}}+\mu_{\text{DTH}}))^{s=\text{DIS}}\,(\mu_{\text{DTH}}/(\mu_{\text{DIS}}+\mu_{\text{DTH}}))^{s=\text{DTH}}\exp(-(\mu_{\text{DIS}}+\mu_{\text{DTH}})t)\right)^{1-s=\text{ICU}}$$

$$(1-\exp\,(-(\mu_{\text{DIS}}+\mu_{\text{DTH}})\, t))^{s=\text{HOS}}$$

As the data is rounded to the nearest day, we discretized the exponential distributions in the likelihood. All models were fitted at maximum likelihood using the software R.

Supplementary Note 2: Model calibration

The model was calibrated to hospital admission and ICU admission data through a maximum likelihood approach. The likelihood function is of the form

$$L(\mathrm{Data}|\Theta) = \prod_{t=t_1}^{t_n} \mathrm{Poiss}\left(\mathrm{ADM_{obs}}(t)\middle|\mathrm{ADM_{pred}}(t)\right)$$

where Θ indicates the set of parameters to be estimated, $\mathrm{ADM_{obs}}(t)$ is the observed number of hospital/ICU admissions on day t, $\mathrm{ADM_{pred}}(t)$ is the number of hospital/ICU admissions predicted by the model on day t, $\mathrm{Poiss}\left(\cdot\middle|\mathrm{ADM_{pred}}(t)\right)$ is the probability mass function of a Poisson distribution with mean $\mathrm{ADM_{pred}}(t)$, and $[t_1, t_n]$ is the time window considered for the fit. Calibration is performed in two steps. First, we fit the transmission rate per contact before lockdown and the starting date of the simulation, considering hospital admissions occurred between March 1 and March 23, 2020. Hospital admissions in the interval March 17-23 were included as still not affected by lockdown, due to delay between date of infection and date of hospitalization (~1 week). Secondly, we fit the transmission rate per contact during lockdown, considering ICU admission data in the interval April 13-26, 2020, to avoid fluctuations observed after lockdown entered into effect.

Supplementary Note 3: Model parametrization during lockdown and exit phase

Here we describe in more details how the contact matrices modifications have been implemented during the lockdown period and during exit strategies.

Contact patterns during lockdown were informed from a previous work[1]. The contact matrices have been modified according to the implementation and regulations of lockdown in France, i.e. closure of all school levels, closure of all non-essential activities and displacements outside home allowed only for essential work. Contacts at school and during leisure activities were therefore reduced to zero. To account for how many individuals stayed at home because of telework or because they were workers in the job sectors impacted by the pandemic (e.g. employees at restaurants, cinemas, shops, etc.), we used the variation of mobility in the region pre- and during lockdown. This was measured from mobile phone trajectory data thanks to a collaboration with Orange, the main telephone operator in the country. For Île-de-France we found a reduction in the number of displacements of 70%[14]. Contacts at work and on transports were therefore reduced according to this percentage. We considered also a 50% reduction of contacts established by seniors to model a marked social distancing targeting only the age class at higher risk of complications. Physical contacts outside the household during lockdown are removed to account for the adoption of physical distancing.

Contact patterns after lockdown considered a scenario with moderate interventions of social distancing (i.e. a partial (50%) reopening of non-essential activities, protection of seniors through a reduction of 30%[15] of their contacts) coupled with more or less aggressive test-trace-isolate strategy (25% or 50% isolation of infected individuals, through a reduction of 90% of their contacts), with schools closed. Compared to this scenario, several protocols for school reopening were considered, where the key parameter was attendance each week. Given a value of attendance, the number of contacts in the school matrix was modified to account for the attendance of students in each school level. Contact matrices define the average number of contacts established by the age class in the population. Therefore, attendance of 25% in a given school level corresponds to a reduction of 75% in the number of contacts established at school by students belonging to that school level. All scenarios assume that physical contacts are fully restored phasing out lockdown.

Supplementary Note 4: Additional results

4.1. Additional results corresponding to values of younger children relative transmissibility not shown in main paper

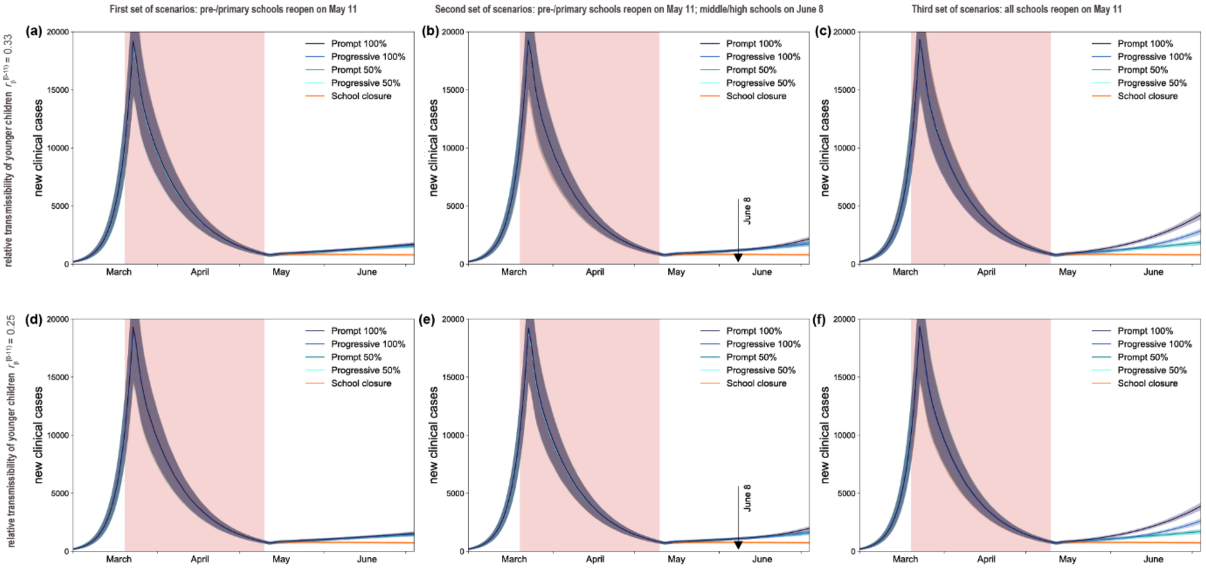

Supplementary Figure 3. Simulated epidemic activity in scenarios with reopening of schools. (a-c) Simulated daily number of new clinical cases assuming that only pre-schools and primary schools are reopened on May 11 through 4 different protocols (first set of scenarios, panel a), additionally considering the reopening of middle and high schools on June 8 (second set of scenarios, panel b), or assuming that all school levels reopen on May 11 (third set of scenarios, panel c). Four protocols (*Progressive (100% ,50%), Prompt (100%, 50%)*) are compared to the school closure scenario. Curves and shaded areas correspond to median and 95% probability ranges, obtained from $n = 500$ independent stochastic runs. Results are obtained for a relative transmissibility of younger children $r_\beta^{[0-11)}$ =0.33. (d-f) As panels (a-c) assuming $r_\beta^{[0-11)}$ =0.25. The red area indicates the lockdown phase. Results are obtained considering moderate social distancing interventions coupled with 50% case isolation.

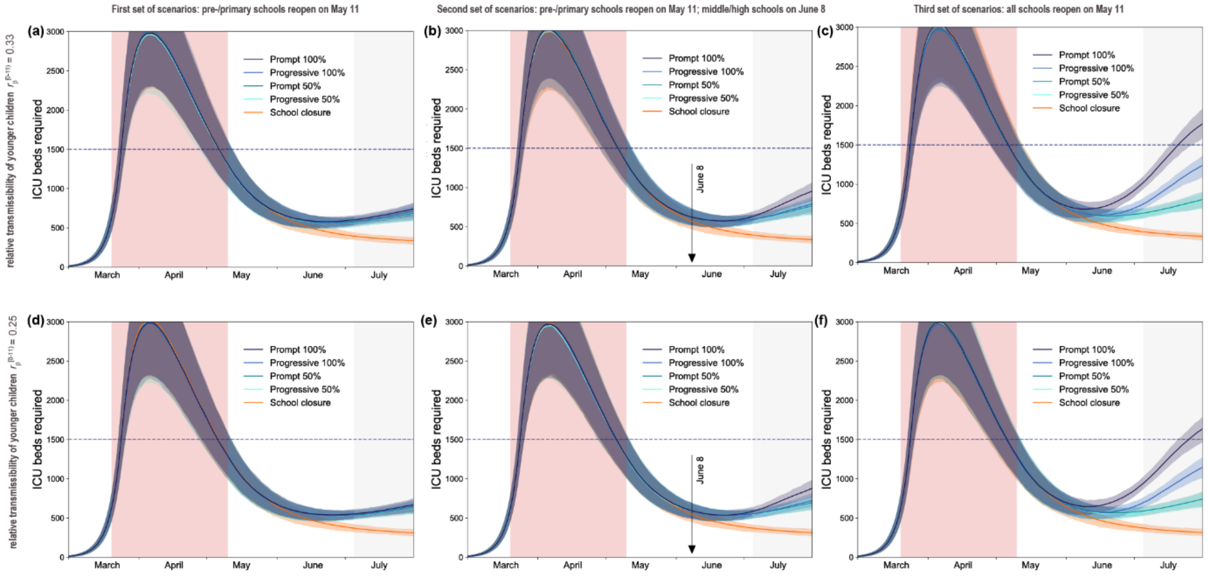

Supplementary Figure 4. Simulated ICU occupancy in scenarios with reopening of schools. (a-c) Simulated demand of ICU beds assuming that only pre-schools and primary schools are reopened on May 11 through 4 different protocols (first set of scenarios, panel a), additionally considering the reopening of middle and high schools on June 8 (second set of scenarios, panel b), or assuming that all school levels reopen on May 11 (third set of scenarios, panel c). Four protocols (*Progressive (100% ,50%), Prompt (100%, 50%)*) are compared to the school closure scenario. Curves and shaded areas correspond to median and 95% probability ranges, obtained from $n = 500$ independent stochastic runs. Results are obtained for a relative transmissibility of younger children $r_\beta^{[0-11)}$ =0.33. (d-f) As panels (a-c) assuming $r_\beta^{[0-11)}$ =0.25. The red area indicates the lockdown phase; the grey area indicates part of summer holidays (month of July to show the delayed effect of the epidemic on ICU demand). Horizontal line refers to the foreseen 1,500-bed ICU capacity in the region restored after the first wave emergency. Results are obtained considering moderate social distancing interventions coupled with 50% case isolation.

Supplementary Note 5: Sensitivity analysis

5.1. Results obtained with an additional set of scenarios

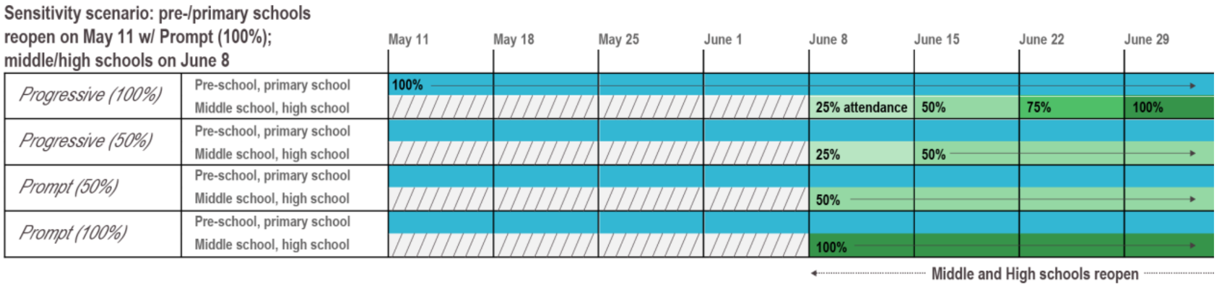

Supplementary Figure 5. Sensitivity scenarios of school reopening. This set of scenarios considers the reopening of pre-schools and primary schools on May 11 through *Prompt (100%)*, followed by the reopening of middle and high schools on June 8 through all 4 possible protocols. This is a variation of the second set of scenarios (Fig. 2). Colors indicate school levels (blue for pre-/primary schools, green for middle/high schools). Color gradient indicate student attendance (from lighter to darker, 25% to 100% at 25% incremental steps).

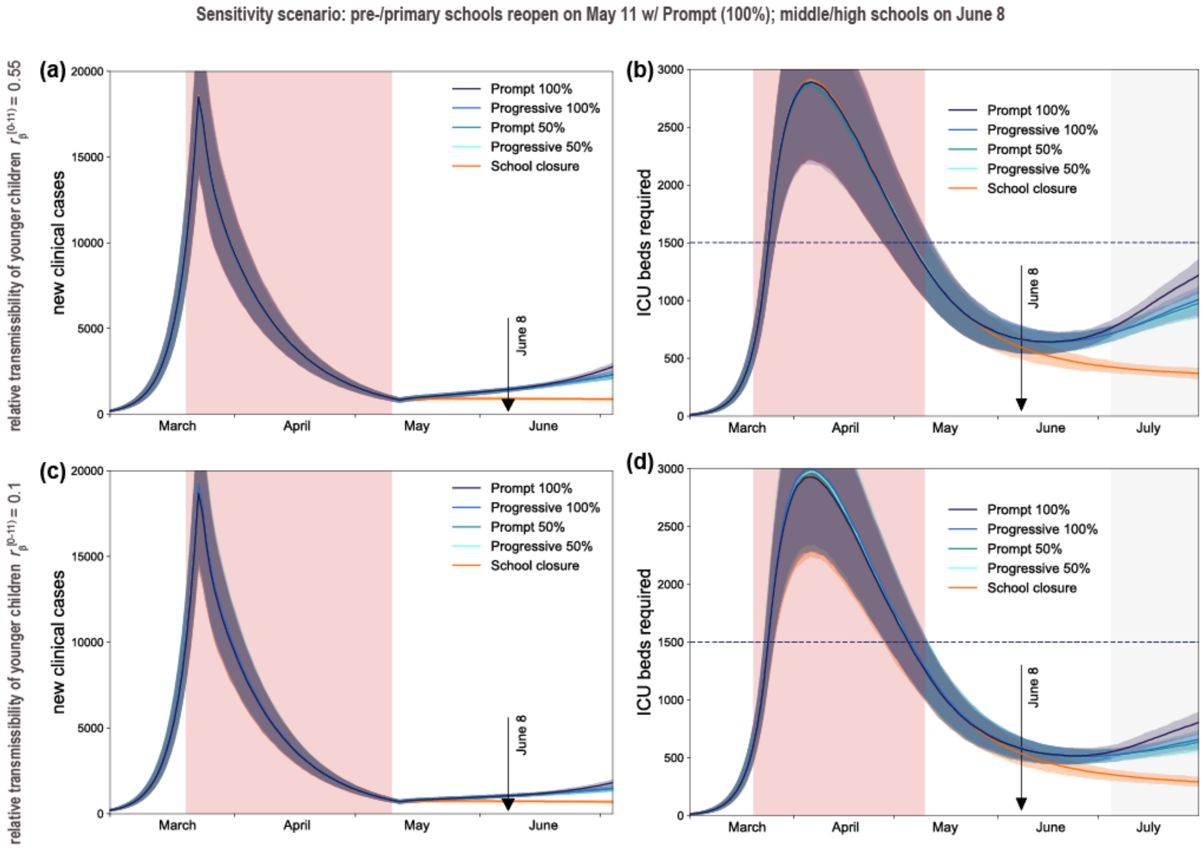

Supplementary Figure 6. Simulated epidemic activity and ICU occupancy in the sensitivity scenarios of school reopening. (a-b) Simulated daily number of new clinical cases (panel a) and simulated demand of ICU beds (panel b) assuming that all pre-schools and primary schools are promptly reopened on May 11, followed by the reopening of middle and high schools on June 8 through four different protocols (*Progressive (100% ,50%), Prompt (100%, 50%)*). Curves and shaded areas correspond to median and 95% probability ranges, obtained from $n = 500$ independent stochastic runs. Results are obtained for a relative transmissibility of younger children $r_\beta^{[0-11)}$ =0.55. (c-d) As (a-b) with $r_\beta^{[0-11)}$ =0.1. The red area indicates the lockdown phase; the grey area indicates summer holidays. Horizontal line in (b-d) refers to the foreseen 1,500-bed ICU capacity in the region restored

after the first wave emergency. Results are obtained considering moderate social distancing interventions coupled with 50% case isolation.

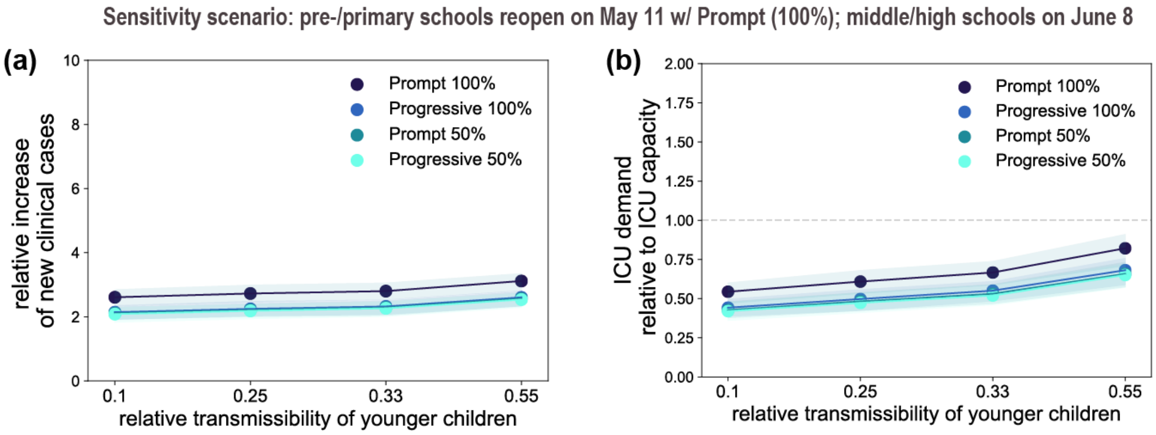

Supplementary Figure 7. Simulated impact of reopening schools in the sensitivity scenarios of school reopening. (a) Projected increase in the daily number of new cases relative to the school closure scenario on July 5 (start of summer holidays) as a function of the relative transmissibility of younger children. (b) Projected ICU demand on August 1 relative to the foreseen 1,500-bed ICU capacity in the region restored after the first wave emergency, as a function of the relative transmissibility of younger children. All pre-schools and primary schools are promptly reopened on May 11, followed by the reopening of middle and high schools on June 8 through four different protocols (*Progressive (100% ,50%), Prompt (100%, 50%)*). Results are obtained considering moderate social distancing interventions coupled with 50% case isolation. Shaded areas correspond to 95% probability ranges around the median value, obtained from $n = 500$ independent stochastic runs.

5.2. Results obtained with 10% variations of the reproduction number during lockdown

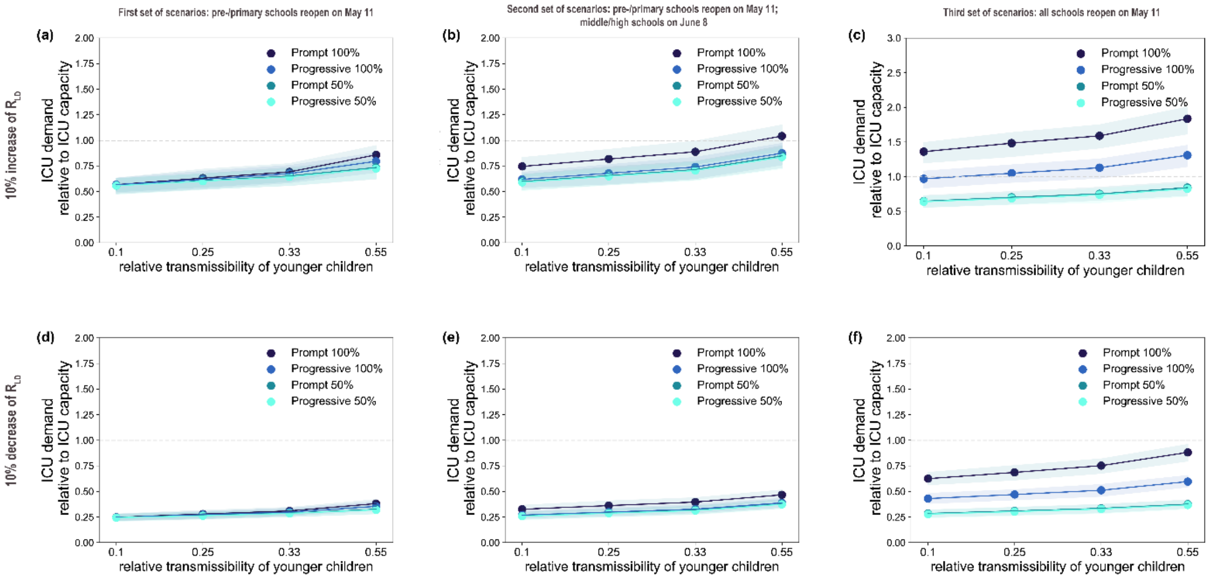

Supplementary Figure 8. Simulated impact of reopening schools on ICU occupancy for 10% variations of the reproduction number. (a-c) Projected ICU demand on August 1 relative to the foreseen 1,500-bed ICU capacity in the region restored after the first wave emergency, as a function of the relative transmissibility of younger children, for different reopening protocols. Results are obtained considering a 10% increase of the reproduction number during lockdown compared to estimate. (d-f) As (a-c) considering a 10% reduction of the reproduction number during lockdown compared to estimate. All results are obtained considering moderate social distancing interventions coupled with 50% case isolation. Shaded areas correspond to 95% probability ranges around the median value, obtained from $n = 500$ independent stochastic runs.

5.3. Results obtained with moderate interventions and 25% case isolation

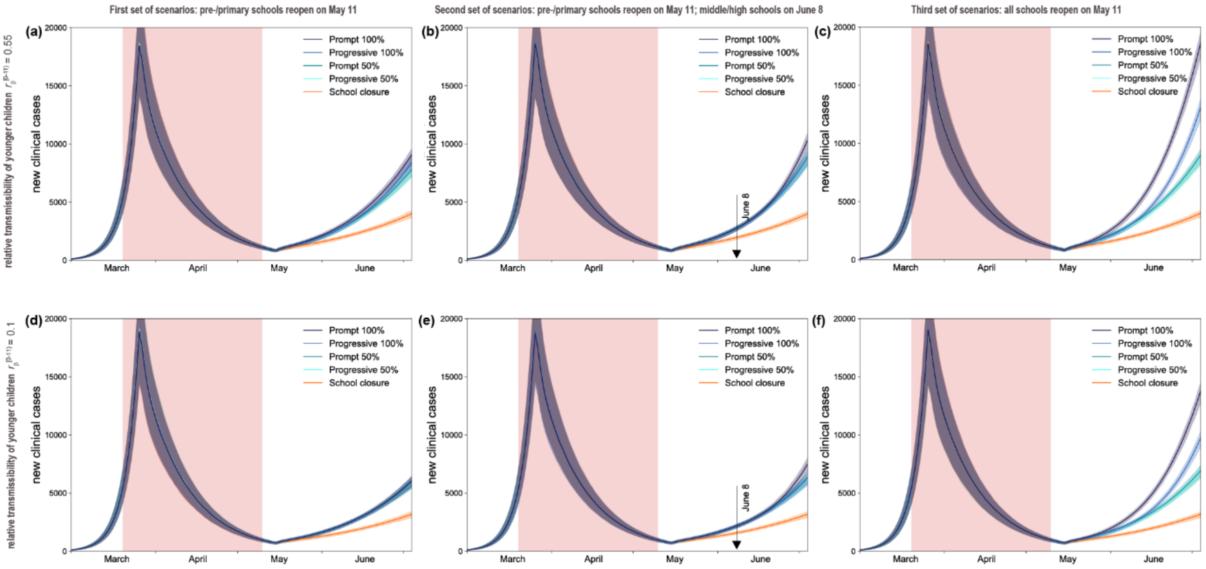

Supplementary Figure 9. Simulated epidemic activity in scenarios with reopening of schools with 25% case isolation. (a-c) Simulated daily number of new clinical cases assuming that only pre-schools and primary schools are reopened on May 11 through 4 different protocols (first set of scenarios, panel a), additionally considering the reopening of middle and high schools on June 8 (second set of scenarios, panel b), or assuming that all school levels reopen on May 11 (third set of scenarios, panel c). Four protocols (*Progressive (100% ,50%), Prompt (100%, 50%)*) are compared to the school closure scenario. Curves and shaded areas correspond to median and 95% probability ranges, obtained from $n = 500$ independent stochastic runs. Results are obtained for a relative transmissibility of younger children $r_{\beta}^{[0-11)}$ =0.55. (d-f) As (a-c) with $r_{\beta}^{[0-11)}$ =0.1. The red area indicates the lockdown phase. Results are obtained considering moderate social distancing interventions coupled with 25% case isolation.

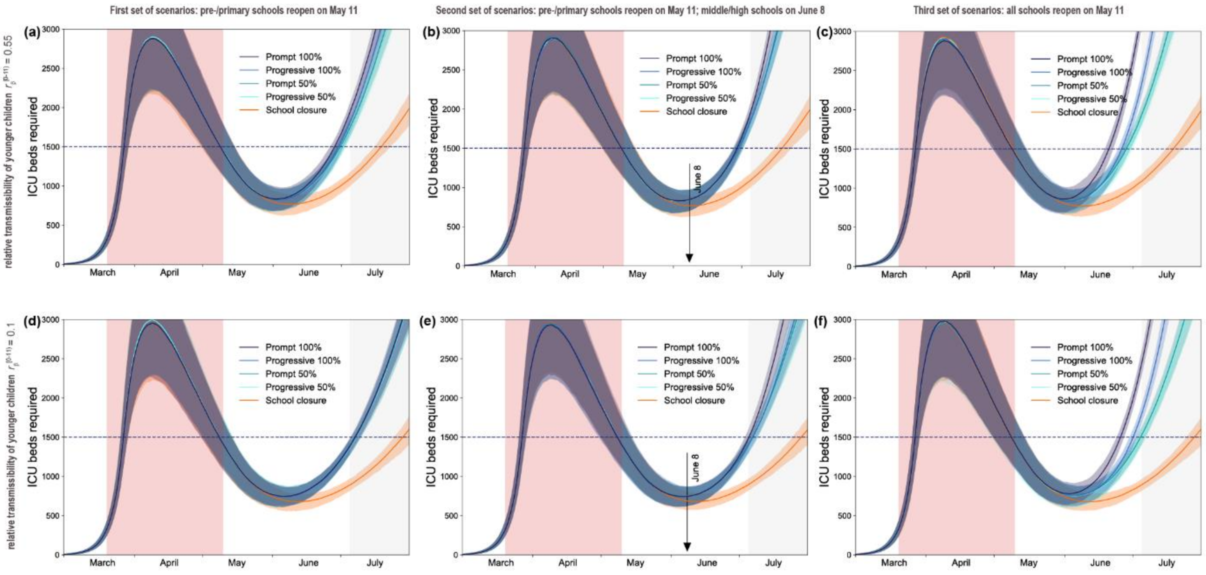

Supplementary Figure 10. Simulated ICU occupancy in scenarios with reopening of schools with 25% case isolation. (a-c) Simulated demand of ICU beds assuming that only pre-schools and primary schools are reopened on May 11 through 4 different protocols (first set of scenarios, panel a), additionally considering the reopening of middle and high schools on June 8 (second set of scenarios, panel b), or assuming that all school levels reopen on May 11 (third set of scenarios, panel c). Four protocols (*Progressive (100% ,50%), Prompt (100%, 50%)*) are compared to the school closure scenario. Curves and shaded areas correspond to median and

95% probability ranges, obtained from $n = 500$ independent stochastic runs. Results are obtained for a relative transmissibility of younger children $r_\beta^{[0-11)}$ =0.55. (d-f) As in (a-c) for $r_\beta^{[0-11)}$ =0.55. The red area indicates the lockdown phase; the grey area indicates summer holidays (month of July to show the delayed effect of the epidemic on ICU demand). Horizontal line refers to the foreseen 1,500-bed ICU capacity in the region restored after the first wave emergency. Results are obtained considering moderate social distancing interventions coupled with 25% case isolation.

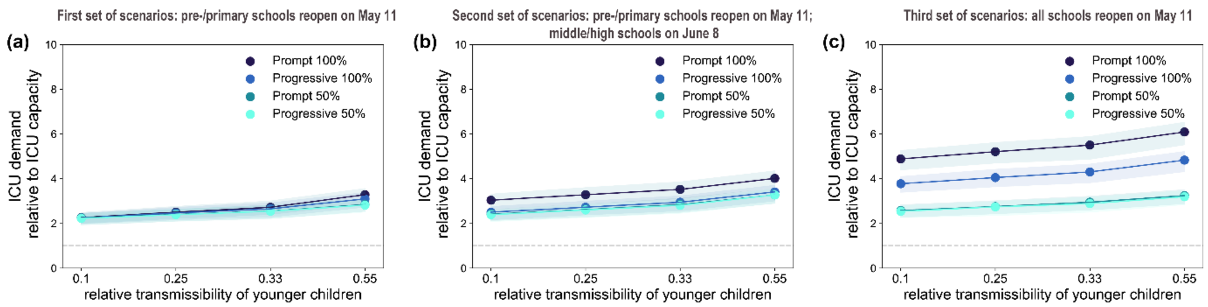

Supplementary Figure 11. Simulated impact on ICU occupancy of reopening schools with 25% case isolation. (a-c) Projected ICU demand on August 1 relative to the foreseen 1,500-bed ICU capacity in the region restored after the first wave emergency, as a function of the relative transmissibility of younger children, for different reopening protocols. Results are obtained considering moderate social distancing interventions coupled with 25% case isolation. Shaded areas correspond to 95% probability ranges around the median value, obtained from $n = 500$ independent stochastic runs.

5.4. Results obtained with 50% reduction of contacts engaged by adolescents

A recent survey conducted in the UK within the BBC pandemic project[16] suggested a decrease of nearly 50% in the average number of contacts made by teenagers (13–18 years) compared with the POLYMOD data, a large-scale endeavour for collecting social contact data in 8 European countries conducted between 2005 and 2006[17]. It is still difficult to interpret these differences. They may genuinely indicate a change in social contacts of teens in the last decade, or they may also be the result of different data collection procedures. For example, in the most recent survey, individuals below 13 years old were not included in the study. To fully answer this question, the same experiment as POLYMOD should be repeated with the same data collection protocol to have a robust assessment of possible changes. Clearly, social mixing conditions are now altered by the pandemic and by the recommendations on the use of preventive measures, such as physical distancing, avoiding physical contacts, avoiding crowded places etc.

In our study, we considered the changes occurring in the population demographic profile from the time of the survey to today to account for changes in social contacts, but mixing rates were taken from the survey. This is a standard approach used in modeling studies (see e.g. Refs.[18,19]). To account for the possible reduction in contacts in teens suggested by the BBC Pandemic study, we assessed the sensitivity of our results considering a baseline contact matrix with 50% reduction in the number of contacts engaged by individuals in the [11,19) age class. Fewer contacts established by adolescents lead to two effects: first, the calibration of the model on the observed epidemic in the pre-lockdown phase leads to a higher transmission rate per contact; second, adolescents play a less important role in the transmission dynamics relatively to the other age classes. Overall, the risk of the reopening of middle and high schools will be less pronounced (Supplementary Figure 12) compared to the results of the main analysis.

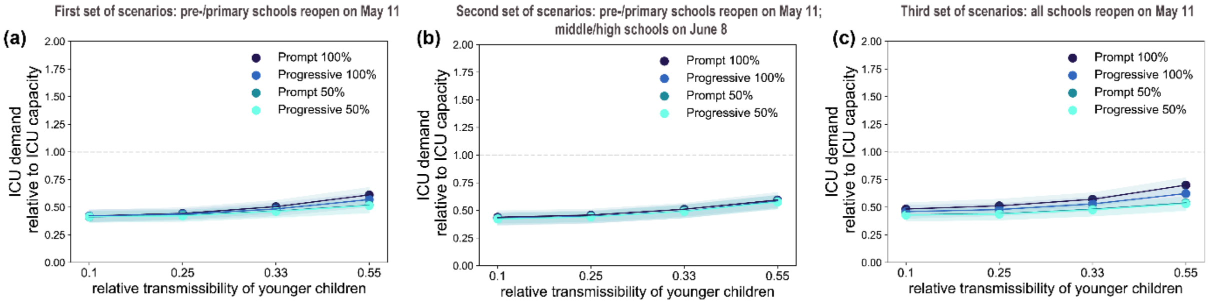

Supplementary Figure 12. Simulated impact on ICU occupancy of reopening schools with 50% reduction of contacts in the [11,19) age class. (a-c) Projected ICU demand on August 1 relative to the foreseen 1,500-bed ICU capacity in the region restored after the first wave emergency, as a function of the relative transmissibility of younger children, for different reopening protocols. Results are obtained considering moderate social distancing interventions coupled with 50% case isolation. Shaded areas correspond to 95% probability ranges around the median value, obtained from $n = 500$ independent stochastic runs.

Supplementary References

1. Di Domenico, L., Pullano, G., Sabbatini, C. E., Boëlle, P.-Y. & Colizza, V. Impact of lockdown on COVID-19 epidemic in Île-de-France and possible exit strategies. *BMC Medicine* **18**, 240 (2020).
2. Pullano, G. *et al.* Underdetection of COVID-19 cases in France threatens epidemic control. *Nature* (2020) doi:10.1038/s41586-020-03095-6.
3. Lauer, S. A. *et al.* The Incubation Period of Coronavirus Disease 2019 (COVID-19) From Publicly Reported Confirmed Cases: Estimation and Application. *Ann Intern Med* **172**, 577 (2020).
4. Ferretti, L. *et al.* Quantifying SARS-CoV-2 transmission suggests epidemic control with digital contact tracing. *Science* (2020) doi:10.1126/science.abb6936.
5. Lavezzo, E. *et al.* Suppression of a SARS-CoV-2 outbreak in the Italian municipality of Vo'. *Nature* **584**, 425–429 (2020).
6. Riccardo, F. *et al.* Epidemiological characteristics of COVID-19 cases in Italy and estimates of the reproductive numbers one month into the epidemic. *Eurosurveillance* **25** (2020).
7. Verity, R. *et al.* Estimates of the severity of coronavirus disease 2019: a model-based analysis. *The Lancet Infectious Diseases*, (2020).
8. Salje, H. *et al.* Estimating the burden of SARS-CoV-2 in France. *Science* **369**, 208–211 (2020).
9. Cereda, D. *et al.* The early phase of the COVID-19 outbreak in Lombardy, Italy. Preprint at http://arxiv.org/abs/2003.09320 (2020).
10. Li, R. *et al.* Substantial undocumented infection facilitates the rapid dissemination of novel coronavirus (SARS-CoV2). *Science* (2020) doi:10.1126/science.abb3221.
11. Davies, N. G. *et al.* Age-dependent effects in the transmission and control of COVID-19 epidemics. *Nature Medicine* **26**, 1205–1211 (2020).
12. Données hospitalières relatives à l'épidémie de COVID-19. https://www.data.gouv.fr/fr/datasets/donnees-hospitalieres-relatives-a-lepidemie-de-covid-19/.
13. Svensson, Å. A note on generation times in epidemic models. *Mathematical Biosciences* **208**, 300–311 (2007).
14. Pullano, G., Valdano, E., Scarpa, N., Rubrichi, S. & Colizza, V. Evaluating the effect of demographic factors, socioeconomic factors, and risk aversion on mobility during the COVID-19

epidemic in France under lockdown: a population-based study. *The Lancet Digital Health* **2**, e638–e649 (2020).

15. Santé Publique France. Covid-19 : une enquête pour suivre l'évolution des comportements et de la santé mentale pendant l'épidémie. /etudes-et-enquetes/covid-19-une-enquête-pour-suivre-l'évolution-des-comportements-et-de-la-sante-mentale-pendant-l-epidemie.
16. Klepac, P. *et al.* Contacts in context: large-scale setting-specific social mixing matrices from the BBC Pandemic project. Preprint at https://www.medrxiv.org/content/10.1101/2020.02.16.20023754v2
17. Mossong, J. *et al.* Social contacts and mixing patterns relevant to the spread of infectious diseases. *PLoS Med.* **5**, e74 (2008).
18. De Luca, G. *et al.* The impact of regular school closure on seasonal influenza epidemics: a data-driven spatial transmission model for Belgium. *BMC Infectious Diseases* **18**, 29 (2018).
19. Arregui, S., Aleta, A., Sanz, J. & Moreno, Y. Projecting social contact matrices to different demographic structures. *PLOS Computational Biology* **14**, e1006638 (2018).
